# Combined pretreatment neutrophil-lymphocyte ratio and platelet-lymphocyte ratio predicts survival and prognosis in patients with non-metastatic nasopharyngeal carcinoma: a retrospective study

**DOI:** 10.1038/s41598-024-59131-2

**Published:** 2024-04-30

**Authors:** Dong Yang, Pian Li, Zhen Meng, Xueying Hu, Zichong Huang, Heqing Huang, Huan Dong, Yating Qin, Cong Chen, Xinghua Chen, Zhiru Li, Ziyan Zhou, Yi Li, Min Kang

**Affiliations:** 1 Department of Radiation Oncology, The First Affiliated Hospital of Guangxi Medical University, Nanning, 530021 Guangxi China; 2 Department of Oncology, The First Affiliated Hospital, Hengyang Medical School, University of South China, Hengyang, 421001 Hunan China; 3 Guangxi Key Laboratory of Immunology and Metabolism for Liver Diseases, Nanning, 530021 Guangxi China; 4 Key Laboratory of Early Prevention and Treatment for Regional High Frequency Tumor (Guangxi Medical University), Ministry of Education, Nanning, 530021 Guangxi China; 5 Department of Obstetrics and Gynecology, The First Affiliated Hospital, Hengyang Medical School, University of South China, Hengyang, 421001 Hunan China; 6 Department of Oncology, Langdong Hospital of Guangxi Medical University, Nanning, 530028 Guangxi China; 7 Department of Clinical Laboratory Medicine, Institution of Microbiology and Infectious Diseases, The First Affiliated Hospital, Hengyang Medical School, University of South China, Hengyang, 421001 Hunan China

**Keywords:** Neutrophil-lymphocyte ratio, Platelet-lymphocyte ratio, Survival, Prognosis, Non-metastatic nasopharyngeal carcinoma, Head and neck cancer, Cancer

## Abstract

The clinical significance of the combination of neutrophil–lymphocyte ratio (NLR) and platelet-lymphocyte ratio (PLR) is unclear. This study investigated the predictive value of pretreatment NLR (pre-NLR) combined with pretreatment PLR (pre-PLR) for the survival and prognosis of nasopharyngeal carcinoma (NPC). A total of 765 patients with non-metastatic NPC from two hospitals were retrospectively analyzed. The pre-NLR-PLR groups were as follows: HRG, high pre-NLR and high pre-PLR. MRG, high pre-NLR and low pre-PLR or low pre-NLR and high pre-PLR. LRG, neither high pre-NLR nor high pre-PLR. Receiver operating characteristic (ROC) curves were used to identify the cutoff-value and discriminant performance of the model. We compared survival rates and factors affecting the prognosis among different groups. The 5-year overall survival (OS), local regional recurrence-free survival (LRRFS) and distant metastasis-free survival (DMFS) of NPC patients in HRG were significantly poorer than those in MRG and LRG. The pre-NLR-PLR score was positively correlated with T stage, clinical stage, ECOG, and pathological classification. Multivariate cox regression analysis showed that pre-NLR-PLR scoring system, ECOG, pre-ALB, pre-CRP and pre-LMR were independent risk factors affecting 5-year OS, LRRFS and DMFS. The ROC curve showed that area under the curve (AUC) values of pre-NLR-PLR of 5-year OS, LRRFS and DMFS were higher than those of pre-NLR and pre-PLR. pre-NLR-PLR is an independent risk factor for the prognosis of NPC. The pre-NLR-PLR scoring system can be used as an individualized clinical assessment tool to predict the prognosis of patients with non-metastatic NPC more accurately and easily.

## Introduction

Nasopharyngeal carcinoma (NPC) occurs all over the world, but there is an obvious regional high incidence phenomenon. Worldwide, NPC has a high incidence in China and Southeast Asian countries, followed by North Africa, while the incidence of NPC is extremely low in Europe, the United States, and Oceania^1^. In China, the incidence of NPC shows a trend of higher in the south and lower in the north, the provinces with high incidence are in the south and southwest of China, such as Guangdong, Guangxi, Hainan, Hong Kong, Macao and Jiangxi. The male to female incidence ratio of NPC is 2.5:1, and the peak age of incidence is 40-59 years old^2^. In recent years, with the development of intensity modulated radiotherapy (IMRT), and the improvement of comprehensive treatment level, the local control rate and survival rate of NPC have been significantly improved, the 5-year survival rate is more than 80%^3^. However, the distant metastasis control rate is still not very ideal, local recurrence and distant metastasis are the main causes of treatment failure in NPC. At present, TNM staging is widely used in the treatment decision and prognosis evaluation of NPC, but there are cases with the same clinical stage but different prognosis, which suggests that there may be tumor biological heterogeneity in NPC patients^4^. Therefore, it is of great significance to use scientific and reasonable means to find reliable and economical prognostic indicators or systems for more accurate prognostic evaluation of NPC.

Recent studies have found that inflammation plays an important role in the occurrence, proliferation, invasion and metastasis of tumors by regulating immunity and promoting angiogenesis, high expression of inflammatory factors is associated with poor prognosis^5^. Systemic inflammation, including neutrophil-to-lymphocyte ratio (NLR), platelet-to-lymphocyte ratio (PLR), lymphocyte-to-monocyte ratio (LMR) and C-reactive protein (CRP), can be used as an independent prognostic biomarker for a variety of tumors^6–8^. Some studies have shown that NLR and PLR have an impact on the prognosis of NPC^9^, but the results are still controversial. Consequently, based on the data of 765 patients with non-metastatic NPC, the aim of this study was to investigate the predictive value of pretreatment NLR (pre-NLR) combined with pretreatment PLR (pre-PLR) for the survival and prognosis of NPC.

## Results

### General clinical features of patients and their correlation with NLR and PLR

A total of 765 patients were enrolled in this study, including 573 males and 192 females, with a median age of 47 years (IQR 40–54 years). Detailed general clinical characteristics were shown in Table 1.

**Table 1:** The 765 NPC patients with general clinical features and the correlation between pre-NLR and pre-PLR groups.

Characteristics	Total (%)	pre-NLR (%)		*p*-Value	pre-PLR (%)		*p*-Value
		Low (n = 484)	High (n = 281)		Low (n = 446)	High (n = 319)	
Gender				0.548			0.007*
Male	573 (74.9)	366 (75.6)	207 (73.7)		350 (78.5)	223 (69.9)	
Female	192 (25.1)	118 (24.4)	74 (26.3)		96 (21.5)	96 (30.1)	
Age (years old)				0.870			0.325
< 47	370 (48.4)	233 (48.1)	137 (48.8)		209 (46.9)	161 (50.5)	
≥ 47	395 (51.6)	251 (51.9)	144 (51.2)		237 (53.1)	158 (49.5)	
8th T stage				0.002*			< 0.001*
T1-T2	133 (17.4)	101 (20.9)	32 (11.4)		96 (21.5)	37 (11.6)	
T3	308 (40.2)	191 (39.5)	117 (41.6)		185 (41.5)	123 (38.6)	
T4	324 (42.4)	192 (39.6)	132 (47.0)		165 (37.0)	159 (49.8)	
8th N stage				0.778			0.192
N0-N1	213 (27.8)	139 (28.7)	74 (26.3)		126 (28.3)	87 (27.3)	
N2	432 (56.5)	270 (55.8)	162 (57.7)		259 (58.1)	173 (54.2)	
N3	120 (15.7)	75 (15.5)	45 (16.0)		61 (13.6)	59 (18.5)	
8th Clinical stage				0.043*			< 0.001*
I-II	37 (4.8)	29 (6.0)	8 (2.8)		29 (6.5)	8 (2.5)	
III	323 (42.3)	212 (43.8)	111 (39.5)		210 (47.1)	113 (35.4)	
IVa	405 (52.9)	243 (50.2)	162 (57.7)		207 (46.4)	198 (62.1)	
ECOG				0.004*			0.004*
0	566 (74.0)	375 (77.5)	191 (68.0)		347 (77.8)	219 (68.7)	
1	199 (26.0)	109 (22.5)	90 (32.0)		99 (22.2)	100 (31.3)	
Pathological type				< 0.001*			0.008*
NKUC	743 (97.1)	478 (98.8)	265 (94.3)		440 (98.6)	303 (95.0)	
NKDC	14 (1.8)	2 (0.4)	12 (4.3)		3 (0.7)	11 (3.4)	
KSCC	8 (1.1)	4 (0.8)	4 (1.4)		3 (0.7)	5 (1.6)	
Smoking				0.232			0.124
No	516 (67.5)	319 (65.9)	197 (70.1)		291 (65.2)	225 (70.5)	
Yes	249 (32.5)	165 (34.1)	84 (29.9)		155 (34.8)	94 (29.5)	
Drinking				0.899			0.388
No	638 (85.6)	415 (85.7)	240 (85.4)		386 (86.5)	269 (84.3)	
Yes	110 (14.4)	69 (14.3)	41 (14.6)		60 (13.5)	50 (15.7)	
Treatment				0.812			0.105
IMRT	43 (5.6)	29 (6.0)	14 (5.0)		28 (6.3)	15 (4.7)	
CCRT	220 (28.8)	137 (28.3)	83 (29.5)		139 (31.2)	81 (25.4)	
CCRT + IC/AC	502 (65.6)	318 (65.7)	184 (65.5)		279 (62.5)	223 (69.8)	
pre-HGB				< 0.001*			< 0.001*
< 120 g/L	212 (27.7)	97 (20.0)	115 (40.9)		67 (15.0)	145 (45.5)	
≥ 120 g/L	553 (72.3)	387 (80.0)	166 (59.1)		379 (85.0)	174 (54.5)	
pre-ALB				0.002*			0.004*
< 43 g/L	354 (46.3)	203 (41.9)	151 (53.7)		187 (41.9)	167 (52.4)	
≥ 43 g/L	411 (53.7)	281 (58.1)	130 (46.3)		259 (58.1)	152 (47.6)	
pre-LMR				< 0.001*			< 0.001*
< 2.82	374 (48.9)	188 (38.8)	186 (66.2)		154 (34.5)	220 (69.0)	
≥ 2.82	391 (51.1)	296 (61.2)	95 (33.8)		292 (65.5)	99 (31.0)	
pre-CRP				< 0.001*			< 0.001*
< 10 mg/L	554 (72.4)	384 (79.3)	170 (60.5)		350 (78.5)	204 (63.9)	
≥ 10 mg/L	211 (27.6)	100 (20.7)	110 (39.5)		96 (21.5)	115 (36.1)	

*NLR* neutrophil-to-lymphocyte ratio, *PLR* platelet-to-lymphocyte ratio, *ECOG* Eastern Cooperative Oncology Group, *IMRT* intensity modulated radiotherapy, *CCRT* concurrent chemoradiotherapy, *IC* induction chemotherapy, *AC* adjuvant chemotherapy, *NKUC* undifferentiated non-keratinizing carcinomas, *NKDC* differentiated non-keratinizing carcinomas, *KSCC* keratinizing squamous cell carcinoma, *HGB* hemoglobin, *ALB* albumin, *CRP* C-reactive protein, *pre* pretreatment.

*Indicates a significant difference among groups with *p* < 0.05.

According to the ROC curve, the cut-off values of pre-NLR, pre-PLR and pre-LMR were 3.29 (AUC 0.661, P < 0.001), 196.74 (AUC 0.651, P < 0.001) and 2.82 (AUC 0.664, P < 0.001) respectively. 63.3% (484/765) of patients had pre-NLR < 3.29 (low grade group) and 36.7% (281/765) had pre-NLR ≥ 3.29 (high grade group). 58.3% (446/765) of patients had pre-PLR < 196.74 (low grade group) and 41.7% (319/765) had pre-PLR ≥ 196.74 (high grade group). 48.9% (374/765) of patients had pre-LMR < 2.82 (low grade group) and 51.1% (391/765) had pre-LMR < 2.82 (high grade group). T stage, clinical stage, ECOG score, pathological classification, pre-HGB, pre-ALB, pre-CRP and pre-LMR were correlated with the level of pre-NLR, and the difference was statistically significant. Gender, T stage, clinical stage, ECOG score, pathological classification, pre-HGB, pre-ALB, pre-CRP and pre-LMR were correlated with the level of pre-PLR, and the differences were statistically significant (Table 1).

### Correlation analysis between general clinical features and pre-NLR-PLR score system

In order to analyze the prognostic value of pre-NLR combined with pre-PLR, the pre-NLR-PLR scoring system was established in this study. Patients were divided into low-risk group (LRG), medium-risk group (MRG) and high-risk group (HRG), pre-NLR-PLR scoring criteria and grouping were as follows: HRG (n = 224, 29.3%), score of 2, high pre-NLR (≥ 3.29) and high pre-PLR (≥ 196.74). MRG (n = 152, 19.9%), score of 1, high pre-NLR and low pre-PLR or low pre-NLR and high pre-PLR. LRG (n = 389,50.8%), score of 0, neither high pre-NLR nor high pre-PLR. The correlation between different risk groups and the general clinical characteristics of patients was shown in Table 2. LRG and MRG had significant differences in T stage (P = 0.001), clinical stage (P < 0.001), pre-HGB (P < 0.001) and pre-LMR (P < 0.001). There were significant differences between LRG and HRG in T stage (P < 0.001), clinical stage (P = 0.002), ECOG score (P = 0.001), pathological classification (P = 0.001), pre-HGB (P < 0.001), pre-ALB (P = 0.001), pre-CRP (P < 0.001) and pre-LMR (P < 0.001). There was significant difference between MRG and HRG in pathological classification (P = 0.023), pre-HGB (P < 0.001), pre-CRP (P = 0.001) and pre-LMR (P < 0.001). There were no significant differences in LRG, MRG and HRG among different genders, ages, N stages, treatment methods, smoking and drinking history.

**Table 2:** Correlation between general clinical features and pre-NLR-PLR groups.

Characteristics	LRG (%) (n = 389)	MRG (%) (n = 152)	HRG (%) (n = 224)	p-Value		
				LRG vs. MRG	LRG vs. HRG	MRG vs. HRG
Gender				0.174	0.073	0.842
Male	303 (77.9)	110 (72.4)	160 (71.4)			
Female	86 (22.1)	42 (27.6)	64 (28.6)			
Age (years old)				0.712	0.463	0.357
< 47	186 (47.8)	70 (46.1)	114 (50.9)			
≥ 47	203 (52.2)	82 (53.9)	110 (49.1)			
8th T stage				0.001*	< 0.001*	0.504
T1-T2	89 (22.9)	19 (12.5)	25 (11.2)			
T3	161 (41.4)	54 (35.5)	93 (41.5)			
T4	139 (35.7)	79 (52.0)	106 (47.3)			
8th N stage				0.035*	0.585	0.132
N0-N1	109 (28.0)	47 (30.9)	57 (25.4)			
N2	228 (58.6)	73 (48.0)	131 (58.5)			
N3	52 (13.4)	32 (21.1)	36 (16.1)			
8th Clinical stage				< 0.001*	0.002*	0.231
I-II	26 (6.7)	6 (3.9)	5 (2.2)			
III	187 (48.1)	48 (31.6)	88 (39.3)			
IVa	176 (45.2)	98 (64.5)	131 (58.5)			
ECOG				0.432	0.001*	0.065
0	304 (78.1)	114 (75.0)	148 (66.1)			
1	85 (21.9)	38 (25.0)	76 (33.9)			
Pathological type				1.000	0.001*	0.023*
NKUC	384 (98.7)	150 (98.6)	209 (93.3)			
NKDC	2 (0.5)	1 (0.7)	11 (4.9)			
KSCC	3 (0.8)	1 (0.7)	4 (1.8)			
Smoking				0.690	0.118	0.370
No	254 (65.3)	102 (67.1)	160 (71.4)			
Yes	135 (34.7)	50 (32.9)	64 (28.6)			
Drinking				0.357	0.638	0.651
No	337 (86.6)	127 (83.6)	191 (85.3)			
Yes	52 (13.4)	25 (16.4)	33 (14.7)			
Treatment				0.155	0.578	0.584
IMRT	26 (6.7)	5 (3.3)	12 (5.4)			
CCRT	118 (30.3)	40 (26.3)	62 (27.7)			
CCRT+IC/AC	245 (63.0)	107 (70.4)	150 (66.9)			
pre-HGB				< 0.001*	< 0.001*	< 0.001*
< 120 g/L	60 (15.4)	44 (28.9)	108 (48.2)			
≥ 120 g/L	329 (84.6)	108 (71.1)	116 (51.8)			
pre-ALB				0.042*	0.001*	0.444
< 43 g/L	157 (40.4)	76 (50.0)	121 (54.0)			
≥ 43 g/L	232 (59.6)	76 (50.0)	103 (46.0)			
pre-LMR				< 0.001*	< 0.001*	< 0.001*
< 2.82	132 (33.9)	78 (51.3)	164 (73.2)			
≥ 2.82	257 (66.1)	74 (48.7)	60 (26.8)			
pre-CRP				0.234	< 0.001*	0.001*
< 10 mg/L	310 (79.7)	114 (75.0)	130 (58.0)			
≥ 10 mg/L	79 (20.3)	38 (25.0)	94 (42.0)			

*HRG* high risk group, *MRG* medium risk group, *LRG* low risk group.

*Indicates a significant difference among groups with *p* < 0.05.

Spearman rank correlation was used to analyze the correlation between patients' general characteristics and pre-NLR-PLR score. pre-NLR-PLR score was positively correlated with T stage, clinical stage, ECOG score, pathological classification and pre-CRP (P < 0.001, P < 0.001, P = 0.002, P < 0.001 and P < 0.001, respectively). pre-NLR-PLR score was negatively correlated with pre-HGB, pre-ALB and pre-LMR. The higher the pre-NLR-PLR score, the lower the pre-HGB, pre-ALB and pre-LMR (P < 0.001, P = 0.001 and P < 0.001, respectively). There was no significant correlation between pre-NLR-PLR score and N stage (Supplementary Table S1).

### Survival of different groups in pre-NLR-PLR scoring system

**Figure 1:**
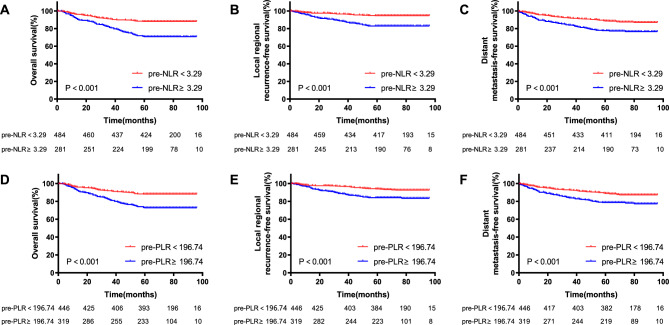
Kaplan-Meier curves of the different ratio of pre-NLR and pre-PLR. The 5-year overall survival rates of pre-NLR (**A**), locoregional recurrence-free survival rates of pre-NLR (**B**), distant metastasis-free survival rates of pre-NLR (**C**), 5-year overall survival rates of pre-PLR (**D**), locoregional recurrence-free survival rates of pre-PLR (**E**) and distant metastasis-free survival rates of pre-PLR (**F**). p values were calculated with the log-rank test.

**Figure 2:**
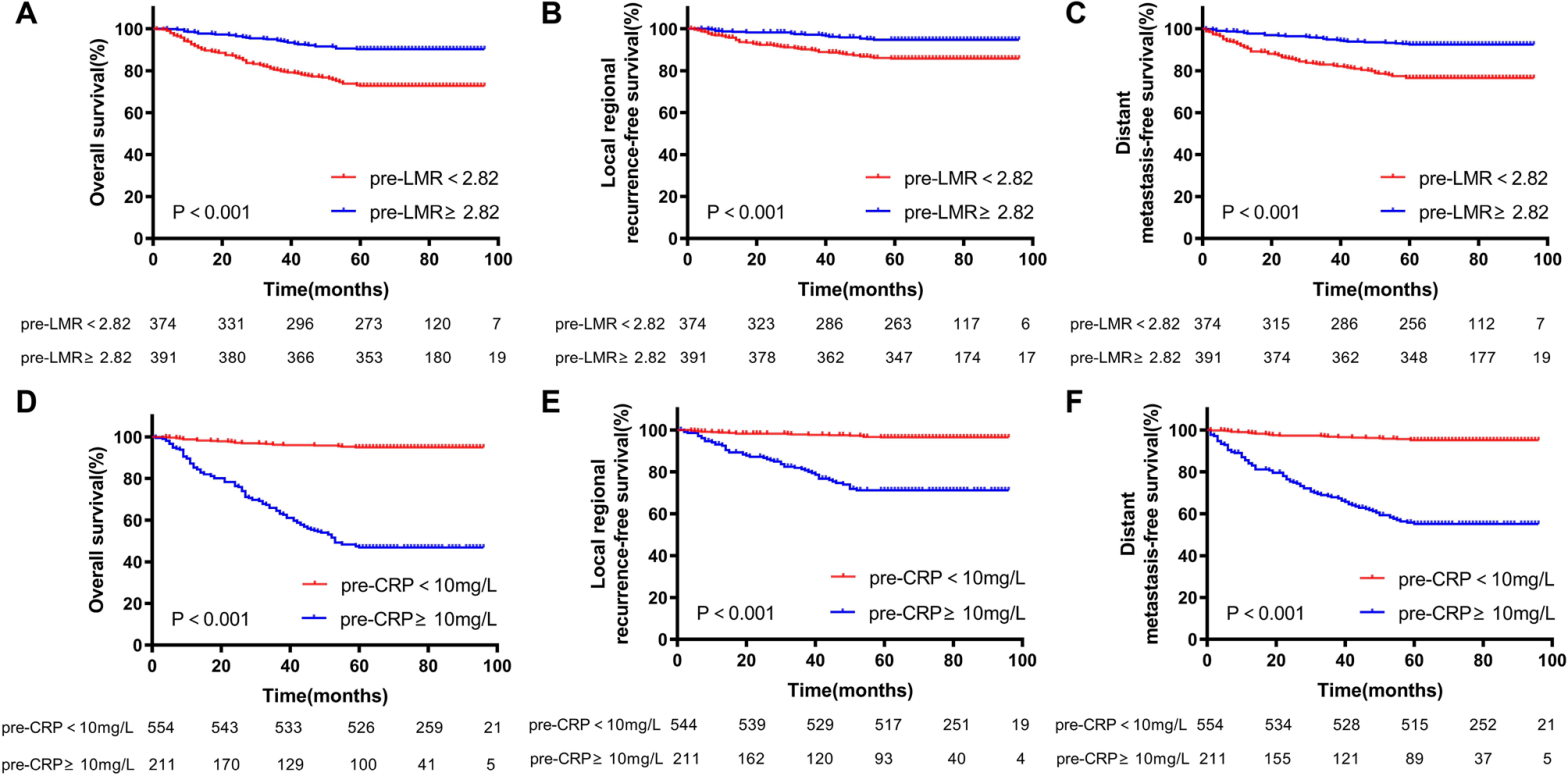
Kaplan-Meier curves of the different ratio of pre-LMR and pre-CRP. The 5-year overall survival rates of pre-LMR (**A**), locoregional recurrence-free survival rates of pre-LMR (**B**), distant metastasis-free survival rates of pre-LMR (**C**), 5-year overall survival rates of pre-CRP (**D**), locoregional recurrence-free survival rates of pre-CRP (**E**) and distant metastasis-free survival rates of pre-CRP (**F**). p values were calculated with the log-rank test.

**Figure 3:**
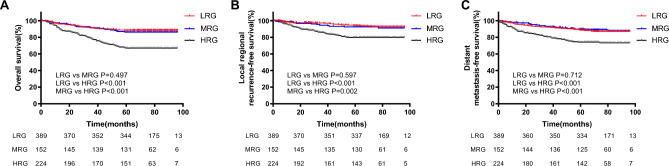
Kaplan-Meier curves of the different ratio of pre-NLR-PLR. The 5-year overall survival rates of pre-NLR-PLR (**A**), locoregional recurrence-free survival rates of pre-NLR-PLR (**B**), distant metastasis-free survival rates of pre-NLR-PLR (**C**). p values were calculated with the log-rank test.

At a median follow-up of 75 months (IQR 64–85 months), a total of 20.4% (156/765) patients died during follow-up period, including 77.6% (121/156) of tumor-related death, and 22.4% (35/156) of other cause. Locoregional recurrence occurred in 79 patients (10.3%), distant metastasis occurred in 122 patients (15.9%), and both locoregional recurrence and distant metastasis occurred in 23 patients. Among the pre-NLR < 3.29 and pre-NLR ≥ 3.29 groups, five-year OS were 88.0% and 70.8%, LRRFS were 94.4% and 83.6%, DMFS were 89.0% and 78.6%, respectively. Among the pre-PLR < 196.74 and pre-PLR ≥ 196.74 groups, five-year OS were 88.1% and 72.7%, LRRFS were 94.32% and 85.3%, DMFS were 89.2% and 79.6%, respectively (Fig. 1). Among the pre-LMR < 2.82 and pre-LMR ≥ 2.82 groups, five-year OS were 72.7% and 90.3%, LRRFS were 86.9% and 94.9%, DMFS were 77.5% and 92.6%, respectively. Among the pre-CRP < 10 mg/L and pre-CRP ≥ 10 mg/L groups, five-year OS were 94.9% and 46.9%, LRRFS were 96.6% and 76.3%, DMFS were 95.1% and 59.2%, respectively (Fig. 2). Among the LRG, MRG and HRG groups, five-year OS were 88.4%, 86.2% and 67.0%, LRRFS were 94.6%, 92.8% and 81.7%, DMFS were 88.9%, 90.1% and 75.4%, respectively. The K-M survival curves of those groups were show in Fig. 3.

### Univariate and multivariate analysis of prognostic factors for NPC

According to univariate cox regression analysis, a number of variables associated with 5-year OS, including age, T stage, N stage, clinical stage, ECOG, pathological type, smoking history, pre-HGB, pre-ALB, pre-LMR, pre-CRP, and pre-NLR-PLR. Variables associated with 5-year LRRFS included age, N stage, ECOG, pathological type, smoking history, pre-ALB, pre-LMR, pre-CRP, and pre-NLR-PLR. Age, T stage, N stage, clinical stage, ECOG, pathological type, pre-HGB, pre-ALB, pre-LMR, pre-CRP, and pre-NLR-PLR were the variables associated with 5-year DMFS (Supplementary Table S2).

Subsequently, multivariate cox regression analysis was conducted for the above variables. Variables for inclusion were carefully chosen, given the number of events available, to ensure parsimony of the final model. For example, because there was no significant difference between LRG and MRG in K-M curve of NPC patients, the pre-NLR-PLR score group was adjusted and included in the multivariate Cox regression analysis. Because the clinical stage was determined by stage T and stage N, we did not include it in the multivariate analysis. Analysis showed that pre-NLR-PLR scoring system, ECOG, pre-ALB, pre-CRP and pre-LMR were independent risk factors affecting 5-year OS, 5-year LRRFS and 5-year DMFS in NPC patients. Among them, Patients with pre-NLR-PLR score of 2 (HRG) were 1.905 times more likely to die within 5 years than those with score of 0–1 (LRG + MRG) (HR 1.905, 95% CI 1.340–2.709, p < 0.001), the risk of local recurrence was 1.864 times more likely than those with LRG + MRG (HR 1.864, 95% CI 1.109–3.133, p = 0.019), and the risk of distant metastasis was 1.631 times than LRG + MRG (HR: 1.631, 95% CI 1.104–2.410, p = 0.014). In addition, age, T(4) stage, N(3) stage and smoking history were independent risk factors for 5-year OS in NPC patients. T stage and N(3) stage were independent risk factors for DMFS at 5 years (Table 3).

**Table 3:** Multivariable analysis of prognostic factors in NPC patients.

Characteristics	5-year OS		5-year LRRFS		5-year DMFS	
	HR (95%CI)	*p*-Value	HR (95%CI)	*p*-Value	HR (95%CI)	*p*-Value
Age (years old)						
< 47	Reference		Reference		Reference	
≥ 47	1.942 (1.348–2.796)	< 0.001*	1.558 (0.928–2.617)	0.093	1.225 (0.834–1.800)	0.300
8th T stage						
T1-T2	Reference		–	–	Reference	
T3	1.744 (0.905–3.360)	0.096	–	–	2.421 (1.140–5.142)	0.021*
T4	1.976 (1.034–3.777)	0.039*	–	–	2.485 (1.170–5.279)	0.018*
8th N stage						
N0-N1	Reference		Reference		Reference	
N2	1.479 (0.936–2.337)	0.094	1.116 (0.597–2.088)	0.731	1.228 (0.757–1.991)	0.405
N3	1.754 (1.012–3.040)	0.045*	1.207 (0.566–2.572)	0.626	2.031 (1.161–3.555)	0.013*
ECOG						
0	Reference		Reference		Reference	
1	9.681 (6.561–14.284)	< 0.001*	4.308 (2.509–7.394)	< 0.001*	6.603 (4.434–9.832)	< 0.001*
Smoking						
No	Reference		Reference		–	–
Yes	1.509 (1.066–2.136)	0.020*	1.507 (0.925–2.455)	0.100	–	–
pre-HGB						
< 120 g/L	Reference		–	–	Reference	
≥ 120 g/L	1.121 (0.771–1.632)	0.550	–	–	1.191 (0.782–1.812)	0.415
pre-ALB						
< 43 g/L	Reference		Reference		Reference	
≥ 43 g/L	0.629 (0.435–0.907)	0.013*	0.552 (0.330–0.924)	0.024*	0.667 (0.445–1.000)	0.049*
pre-CRP						
< 10 mg/L	Reference		Reference		Reference	
≥ 10 mg/L	6.588 (4.213–10.303)	< 0.001*	4.587 (2.564–8.208)	< 0.001*	7.114 (4.444–11.387)	< 0.001*
pre-LMR						
< 2.82	Reference		Reference		Reference	
≥ 2.82	0.443 (0.300–0.654)	< 0.001*	0.515 (0.295–0.897)	0.019*	0.397 (0.255–0.618)	< 0.001*
pre-NLR-PLR						
LRG + MRG (0–1 point)	Reference		Reference		Reference	
HRG (2 point)	1.905 (1.340–2.709)	< 0.001*	1.864 (1.109–3.133)	0.019*	1.631 (1.104–2.410)	0.014*

*Indicates a significant difference among groups with *p* < 0.05.

### Prognostic performance of pre-NLR-PLR scoring system

According to ROC curve analysis, it was concluded that the AUC values of pre-NLR-PLR of 5-year OS, LRRFS and DMFS in NPC patients were higher than those of pre-NLR and pre-PLR. Among them, the difference of the AUC values of pre-NLR-PLR and pre-PLR in 5-year OS and 5-year LRRFS was statistically significant, but not in 5-year DMFS. There was no statistical significance in the AUC values of 5-year OS, LRRFS and DMFS between pre-NLR-PLR and pre-NLR (Table 4).

**Table 4:** Comparison of ROC curves of pre-NLR-PLR, pre-NLR and pre-PLR.

Prognostic factors	5-year OS		5-year LRRFS		5-year DMFS	
	AUC	p-Value	AUC	p-Value	AUC	p-Value
pre-NLR-PLR	0.652	N/A	0.664	N/A	0.609	N/A
pre-NLR	0.634	0.089^a^	0.649	0.272^a^	0.596	0.253^a^
pre-PLR	0.625	**0.008**^b^*	0.629	**0.013**^b^*	0.593	0.121^**b**^

*Indicates a significant difference among groups with p < 0.05.

^a^Comparison between pre-NLR-PLR and pre-NLR.

^b^Comparison between pre-NLR-PLR and pre-PLR.

## Discussion

With the wide application of IMRT and the introduction of comprehensive treatment modalities such as chemoradiotherapy combined with immunotherapy and targeted therapy, the local control rate of NPC patients has been significantly improved, and the 5-year overall survival rate has reached more than 80%^2,12,13^. In spite of this, local recurrence and distant metastasis still remain the leading cause of NPC treatment failure, accounting for about 70% of NPC specific deaths^14^. At present, the prognosis of NPC patients still depends on TNM staging system^15^. Nevertheless, although patients have the same clinical stage and receive the same treatment, there are still different treatment effects, which may be due to tumor heterogeneity, immune and inflammatory responses. TNM staging system is based on imaging and anatomy, and does not take into account the biological diversity of the tumor, so the stratified prognosis cannot be satisfactorily evaluated. Some scholars^16^ proposed to use molecular genetic biomarkers, such as mRNA, as new prognostic predictors. However, the detection process of these markers is complicated and the detection cost is high. Many technologies are limited to the laboratory and cannot be promoted clinically.

Inflammation and immune response are involved in the occurrence and development of tumor, including initiation, promotion and metastasis^17^. Since the first reported by Virchow in 1863^18^, more and more studies have shown that inflammatory marker response plays a key role in tumor development and has shown independent prognostic value, such as NLR^5,17,19^, LMR^20^, PLR^21^. Malignant tumors induce systemic inflammatory responses by releasing cytokines and chemokines, which are manifested as elevated neutrophil and platelet counts and decreased lymphocytes^22^. Chen et al.^23^ found in their study on the prognosis of 299 patients with limits-stage small cell lung cancer after surgery that the high levels of preoperative NLR and PLR indicated poor prognosis of patients with limits-stage small cell lung cancer after surgery, and the detection was simple, fast, and low-cost, which could be used as a reference for initial screening of patients who would benefit from immunotherapy. Previous studies have shown that NLR and PLR are associated with the prognosis of NPC^24,25^. However, no comprehensive evaluation of the relationship between the combination of NLR and PLR and the prognosis of NPC patients has been reported. Therefore, based on the current research results that both NLR and PLR have an impact on the prognosis of NPC, our study combined these two hematological parameters and established pre-NLR-PLR scoring system, divided the NPC patients into groups according to different scores, investigated the correlation between hematological parameters and clinical characteristics, survival and prognosis of patients with NPC. In addition, in this study, NLR, PLR and other indicators closely related to OS, LRRFS, and DMFS were detected only by peripheral blood of patients with NPC, the method was simple, effective, reproducible, and practical.

The level of NLR is closely related to the occurrence and development of tumor. It is speculated that the mechanism may be as follows: (1) neutrophils affect tumor microenvironment by releasing matrix metalloproteinase-9, vascular endothelial growth factor and other factors, promoting tumor neovascularization, and promoting tumor occurrence and development^26^; (2) neutrophils promote tumor movement and migration by releasing enzymes, and promote tumor invasion and metastasis; (3) the rise of neutrophils can inhibit the immune cell activity of lymphocytes, natural killer cells and activated T cells, thus reducing the immunity of the body; (4) as the main members of tumor immunity, lymphocytes are involved in cell destruction and apoptosis. Lymphocytes such as CD4 + and CD8 + T cells can induce tumor cell apoptosis and inhibit tumor progression through immune-mediated cytotoxic activity^27^. In this study, pre-NLR was related to T stage, clinical stage, ECOG, pathological type, pre-HGB, pre-CRP, pre-LMR and pre-ALB of patients with NPC, which was similar to the conclusions of previous studies^28,29^. In addition, in this study, 5-year OS, LRRFS and DMFS of NPC patients with pre-NLR ≥ 3.29 were significantly lower than those with pre-PLR < 3.29, indicating that pre-NLR is a risk factor for survival and prognosis of NPC, high pre-PLR indicates poor prognosis.

Tumor growth requires abundant blood supply, platelets can promote angiogenesis and release growth factors, tumor cells mediate platelet aggregation, leading to the body in a hypercoagulable state. Platelet aggregation around tumor cells can protect them from NK cell killing, regulate the process of tumor micrometastasis by activating TGF-β signal transduction pathway, and promote tumor cell exosmosis^30,31^. Studies have shown that PLR can reflect not only the tumor-promoting state and inflammatory response in the body, but also the anti-tumor immune state. SUN et al.^32^ found that PLR is a prognostic factor affecting PFS and OS in non-metastatic NPC, while some studies^33–36^ believed that PLR is not significantly correlated with survival and prognosis of NPC, which may be attributed to tumor heterogeneity. In this study, pre-PLR was related to the gender, T stage, clinical stage, ECOG, pathological type, pre-HGB, pre-CRP, pre-LMR and pre-ALB of patients with NPC, which was consistent with the conclusion of JIANG et al.^31^ who studied 247 patients with NPC who received IMRT. In addition, 5-year OS, LRRFS and DMFS of NPC patients with pre-PLR ≥ 196.74 were significantly lower than those with pre-PLR < 196.74, indicating that pre-PLR is a risk factor for survival and prognosis of NPC, high pre-PLR indicates poor prognosis.

Most previous studies evaluated NLR, PLR individually as well as their clinical significance in patients with various malignant tumors, including NPC. However, it is one-sided to rely solely on TNM staging and NLR or PLR to assess the prognosis. In the past, some studies have combined NLR and PLR to predict the prognosis of cancer, such as peritoneal metastasis cancer^37^, breast cancer^38^, gastric cancer^21^, etc., but no relevant study has carried out the combination of NLR and PLR to predict the prognosis of NPC. Therefore, this study combined these two hematological indicators to establish the pre-NLR-PLR scoring system innovatively. When comparing the baseline characteristics of patients, significant differences were found in T stage, N stage, clinical stage, ECOG, pathological type, pre-HGB, pre-CRP, pre-LMR and pre-ALB among different groups of patients. Survival analysis showed that 5-year OS, LRRFS, and DMFS in HRG (score of 2) patients were significantly worse than those in MRG (score of 1) and LRG (score of 0). Multivariate analysis showed that pre-NLR-PLR scoring system was an independent prognostic factor for NPC patients. Patients with pre-NLR-PLR score of 2 (HRG) were 1.905 times more likely to die within 5 years than those with score of 0-1 (LRG + MRG) (p < 0.001), the risk of local recurrence was 1.864 times more likely than those with LRG + MRG (p = 0.019), and the risk of distant metastasis was 1.631 times than LRG + MRG (p = 0.014). In ROC curve analysis, the AUC values of pre-NLR-PLR scoring system on OS, LRRFS and DMFS in 5 years were higher than those of pre-NLR and pre-PLR, which also indicated that pre-NLR-PLR scoring system had certain advantages in predicting the prognosis of patients with NPC.

These results may suggest that the pre-NLR-PLR scoring system can discriminate patients with better prognosis after treatment from all patients, compared with pre-NLR or pre-PLR alone, and the pre-NLR-PLR scoring system is a potentially useful prognostic predictor that can be assessed before treatment. Those would be the greatest advantage of the pre-NLR-PLR score. In addition, the pre-NLR-PLR score can be easily determined by calculating the NLR and PLR with a small volume of blood (only 2 mL). Thus, assessment of the pre-NLR-PLR score is inexpensive.

To be honest, our study also has some limitations. Plasma EBV DNA has emerged as an important prognostic factor in contemporary studies, as a relatively new technique, the 5-year survival results of EBV DNA are not yet available at our center, so they were not included in our study. This study is a retrospective study, and all eligible patients have different treatment methods according to the choice of the doctors in charge, including chemotherapy regimen, chemotherapy cycle and drug dosage, which may affect the results of the study. some sample sizes are small, such as NKDC and KSCC, which may also affect the results of the study. In addition, without validation with other datasets, the NLR-PLR (cutoff scores of 3.29 and 196.74) is not sufficient for clinical use as a prognostic predictor of NPC. In the future, it is necessary to expand the sample size, collect the 5-year survival results of EBV DNA, further improve the classification of various clinical factors, control other factors that may affect the results, find and include more factors that may affect the outcome and prognosis, verify the usefulness of the NLR-PLR score by using a validation cohort, and explore a more perfect scoring system for better application in clinical diagnosis and treatment.

## Conclusions

In summary, we established the pre-NLR-PLR scoring system innovatively, which is an independent risk factor for the prognosis of NPC. NPC patients with high pre-PLR and high pre-NLR have poor prognosis. The pre-NLR-PLR scoring system can be used as an individualized clinical assessment tool to predict the prognosis of patients with non-metastatic NPC more accurately and easily.

## Methods

### Patients

We retrospectively analyzed 765 patients with non-distant metastatic NPC treated with IMRT in two hospitals from December 2014 to December 2017 (the First Affiliated Hospital of Guangxi Medical University, 748 patients; the First Affiliated Hospital of University of South China, 17 patients). All patients met the following inclusion criteria: (1) confirmed by histopathology as NPC; (2) there was no distant metastasis before and during treatment; (3) had not received any antitumor therapy before; (4) denied the history related to other malignancies; (5) Eastern Cooperative Oncology Group(ECOG) score 0 ~ 1; (6) received radiotherapy or concurrent chemoradiotherapy with/without induction or adjuvant chemotherapy, and completed the entire treatment as planned; (7) complete clinical data, examination data and follow-up data were available. Our exclusion criteria included: (1) distant metastasis was found or could not be ruled out before and during treatment; (2) complicated with severe infection, underlying diseases; (3) previous or concurrent history of other malignant tumors; (4) unable to complete the treatment.

The data of all NPC patients’ serum biomarkers and clinical characteristics were measured and collected within the two weeks before initiating treatment, including age, gender, pathological type, treatment regimen, smoking history, drinking history, ECOG score, radiotherapy technique and dose, chemotherapy regimen and dose, pretreatment (pre-) blood cell count (lymphocytes, neutrophils, monocytes, platelets, and white blood cells), pre-hemoglobin (pre-HGB), and pre-albumin (pre-ALB). We restaged all patients by the eighth edition of the AJCC/UICC TNM staging system. NLR was determined by dividing the absolute neutrophil count by the absolute lymphocyte count. PLR was determined by the absolute platelet count divided by the absolute lymphocyte count. The Ethics Committee at the First Affiliated Hospital of Guangxi Medical University approved the study, which analyzed anonymous information as well as waived the demand for informed consent (Approval Number: 2023-E207-01). The study was conducted in accordance with relevant guidelines and legislation.

### Therapeutic schedule

In this study, TNM staging was performed according to the guidelines of the National Comprehensive Cancer Network (NCCN), and the standardized treatment plan was determined according to the TNM stage of the patient. Patients with stage I received radical radiotherapy. Patients in stage II were treated with radiotherapy or concurrent chemoradiotherapy combined with platinum. Patients with stage III–IVa were treated with concurrent induction chemotherapy or adjuvant chemotherapy. The patients were all treated with IMRT. The radiotherapy target areas of NPC include gross tumor volume of nasopharynx (GTVnx), metastatic cervical lymph node volume (GTVnd), Two clinical target volumes (CTV1 is high risk clinical target volume, CTV2 is low risk clinical target volume), GTV or CTV expansion of 3 to 5mm is the planned target volume (PTV). According to the nasopharyngeal primary focus, nasopharyngeal subclinical focus, cervical lymph node and cervical lymph drainage area, different prescription doses were given respectively. Prescription dosage of nasopharyngeal primary focus, PTV-GTVnx (68 ~ 76 Gy), PTV-CTV1 (60 ~ 64 Gy), PTV-CTV2 (50 ~ 54 Gy), 5 fractions/week for a total of 30–33 fractions. Prescription dosage of cervical lymph node, PTV-GTVnd (66 ~ 70 Gy), PTV-CTV2 (50 ~ 54 Gy), 5 fractions/week for a total of 30–33 fractions. For tissue and organ limit dose, refer to QUANTEC (2012 standard). Induction or adjuvant chemotherapy mainly included GP regimen (gemcitabine 1000 mg/m^2^ on days 1 and 8 + cisplatin 80 mg/m^2^ on day 1), PF regimen (cisplatin 80 mg/m^2^ on day 1 + 5-fluorouracil 800 ~ 1000 mg/m^2^, continuous intravenous drip from day 1 to day 5), TPF regimen (docetaxel 60 mg/m^2^ on day 1 + cisplatin 60 mg/m^2^ on day 1 + 5-fluorouracil 600 mg/m^2^, continuous intravenous drip from day 1 to day 5), and TP regimen (docetaxel 75 mg/m^2^ on day 1 + cisplatin 75 mg/m^2^ on day 1), once every 3 weeks, for a total of 2 ~ 3 cycles. Concurrent chemotherapy regimen used cisplatin (80 ~ 100 mg/m^2^) once every 3 weeks for 2 ~ 3 cycles, or cisplatin (30 ~ 40 mg/m^2^) once a week for 5 ~ 6 cycles. For patients who were not suitable for cisplatin during chemotherapy, other platinum-based drugs were used instead. Immune checkpoint inhibitors (ICIs) were not used in each case in this cohort. Details of chemotherapy regimens in each group were provided in Supplementary data Table S3.

### Endpoint and follow-up

The primary endpoint of the study was overall survival (OS, defined as calculated from the start of treatment to the date of death from any cause). The secondary endpoints were locoregional recurrence-free survival (LRRFS, defined as calculated from the start of treatment to the date of recorded relapse in the local area) and distant metastasis free survival (DMFS, defined as calculated from the start of the first treatment to the date of recorded distant metastasis). Patients were followed up at least every 3 months for the first 2 years after completion of treatment and at least every 6 months for 2 to 5 years after completion of treatment, or until death. Efficacy evaluation was performed according to RECIST Version 1.1 of the Efficacy Evaluation Criteria for solid tumors. The follow-up period ended on August 30, 2022.

### Statistical analysis

SPSS version 26.0 (IBM Corporation, Armonk, NY, USA) and MedCalc 20.1 (MedCalc Software Ltd, Ostend, Belgium) were used for all statistical analyses. P < 0.05 was considered statistically significant.

We transformed continuous variables into categorical variables. The age was grouped into < 47, and ≥ 47 years old. According to the standard of anemia, pre-HGB was divided into < 120 g/L and ≥ 120 g/L^10^. According to previous studies, pre-ALB was divided into < 43 g/L and ≥ 43 g/L^11^. The cut-off value of CRP was set at 10 mg/L according to previous studies^8^. The general characteristics of patients were compared with frequency and descriptive statistics. A chi-square test or Fisher's exact test was used to compare the characteristics of patients in different groups. To determine the cut-off value of the research indicators based on 5-year OS, Youden index of receiver operating characteristic (ROC) curves were used. The area under the curve (AUC) > 0.5 is considered to be a predictive value. To plot survival curves and compare survival among groups, Kaplan–Meier and Log rank tests were used. The factors with P < 0.1 were selected for multivariate analysis based on univariate cox regression analysis. The multivariate cox regression analysis showed independent risk factors for NPC with P < 0.05. A ROC curve was performed to assess whether NPC prognosis could be accurately predicted by pre-NLR combined with pre-PLR. The area under the curve (AUC) > 0.6 is considered to be a predictive value. Delong test was used to compare the classification efficiency of these ROC curves. P < 0.05 indicated an important statistical significance.

### Ethics approval and consent to participate

This study was conducted in accordance with the Helsinki Declaration and approved by the Ethics Committee of the First Affiliated Hospital of Guangxi Medical University. Participant information is confidential. The need for informed consent was waived by the Ethics Committee of the First Affiliated Hospital of Guangxi Medical University. (Approval Number: 2023-E207-01).

### Supplementary Information

Supplementary Information.

## Data Availability

The datasets used and/or analysed during the current study are available from the corresponding author on reasonable request.

## Abbreviations

NPC: Nasopharyngeal carcinoma
NLR: Neutrophil-to-lymphocyte ratio
PLR: Platelet-to-lymphocyte ratio
LMR: Lymphocyte-to-monocyte ratio
CRP: C-reactive protein
ECOG: Eastern Cooperative Oncology Group
IMRT: Intensity modulated radiotherapy
OS: Overall survival
LRRFS: Locoregional recurrence-free survival
DMFS: Distant metastasis-free survival
ROC: Receiver operating characteristic
AUC: Area under the curve
CCRT: Concurrent chemoradiotherapy
IC: Induction chemotherapy
AC: Adjuvant chemotherapy
HRG: High risk group
MRG: Medium risk group
LRG: Low risk group
NKUC: Undifferentiated non-keratinizing carcinomas
NKDC: Differentiated non-keratinizing carcinomas
KSCC: Keratinizing squamous cell carcinoma
HGB: Hemoglobin
ALB: Albumin
HR: Hazard ratio
CI: Confidence interval
