# Supplementary material for: Combined pretreatment neutrophil-lymphocyte ratio and platelet-lymphocyte ratio predicts survival and prognosis in patients with non-metastatic nasopharyngeal carcinoma: a retrospective study
Source: Sci Rep. 2024 Apr 30;14:9898. doi: 10.1038/s41598-024-59131-2 (PMC11061272; doi:10.1038/s41598-024-59131-2)
Supplement: Supplementary file 1 — Supplementary Information. [file 41598_2024_59131_MOESM1_ESM.docx]

**Supplementary Material**

**Combined pretreatment neutrophil-lymphocyte ratio and platelet-lymphocyte ratio predicts survival and prognosis in patients with non-metastatic nasopharyngeal carcinoma: a retrospective study**

Dong Yang, Yi Li, Pian Li, Zhen Meng, Xueyin Hu, Zichong Huang, Heqing Huang, Huan Dong, Yating Qin, Cong Chen, Xinghua Chen, Zhiru Li, Ziyan Zhou, Min Kang

**Supplementary Table 1**

Correlation analysis between clinical characteristics and pre-NLR-PLR...................... 2

**Supplementary Table 2**

Univariate analysis of prognostic factors in NPC patients............................................. 3

**Supplementary Table 3**

Chemotherapy regimens for different groups................................................................ 5

**Supplementary Table 1** Correlation analysis between clinical characteristics and pre-NLR-PLR

| characteristics | pre-NLR-PLR | |
| --- | --- | --- |
|  | r | p-Value |
| 8^th^ T stage | 0.150 | <0.001* |
| 8^th^ N stage | 0.036 | 0.316 |
| 8^th^ Clinical stage | 0.145 | <0.001* |
| ECOG | 0.114 | 0.002* |
| Pathological type | 0.127 | <0.001* |
| pre-HGB | -0.312 | <0.001* |
| pre-ALB | -0.123 | 0.001* |
| pre-LMR | -0.337 | <0.001* |
| pre-CRP | 0.199 | <0.001* |

* indicates a significant difference among groups with *p* < 0.05.

**Supplementary Table 2** Univariate analysis of prognostic factors in NPC patients

| Characteristics | 5-year OS | | 5-year LRRFS | | | 5-year DMFS | | |
| --- | --- | --- | --- | --- | --- | --- | --- | --- |
|  | HR (95%CI) | *p*-Value | HR (95%CI) | *p*-Value | | HR (95%CI) | | *p*-Value |
| Gender |  |  |  | |  |  |  | |
| Male | Reference |  | Reference | |  | Reference |  | |
| Female | 0.758(0.504-1.141) | 0.184 | 0.737(0.410-1.325) | | 0.308 | 0.703(0.441-1.120) | 0.138 | |
| Age(years old) |  |  |  | |  |  |  | |
| <47 | Reference |  | Reference | |  | Reference |  | |
| ≥47 | 2.201(1.541-3.146) | <0.001* | 2.153(1.297-3.572) | | 0.003* | 0.712(0.490-1.035) | 0.075* | |
| 8^th^ T stage |  |  |  | |  |  |  | |
| T1-2 | Reference |  | Reference | |  | Reference |  | |
| T3 | 2.514(1.322-4.781) | 0.005* | 1.555(0.707-3.424) | | 0.273 | 2.975(1.412-6.270) | 0.004* | |
| T4 | 2.800(1.482-5.291) | 0.002* | 1.898 (0.879-4.100) | | 0.103 | 3.050(1.451-6.408) | 0.003* | |
| 8^th^ N stage |  |  |  | |  |  |  | |
| N0-N1 | Reference |  | Reference | |  | Reference |  | |
| N2 | 1.626(1.047-2.524) | 0.030* | 1.434(0.780-2.636) | | 0.246 | 1.264(0.787-2.029) | 0.332 | |
| N3 | 2.165(1.280-3.660) | 0.004* | 2.003(0.967-4.149) | | 0.062* | 2.287(1.331-3.928) | 0.003* | |
| 8^th^ Clinical stage |  |  |  | |  |  |  | |
| I-II | Reference |  | Reference | |  | Reference |  | |
| III | 1.899(0.592-6.098) | 0.281 | 1.367(0.322-5.799) | | 0.671 | 2.282(0.551-9.459) | 0.255 | |
| IVa | 2.935(0.929-9.273) | 0.067* | 2.184(0.530-9.011) | | 0.280 | 3.670(0.901-14.954) | 0.070* | |
| ECOG |  |  |  | |  |  |  | |
| 0 | Reference |  | Reference | |  | Reference |  | |
| 1 | 11.692(7.964-17.165) | <0.001* | 7.944(4.803-13.140) | | <0.001* | 7.785(5.258-11.525) | <0.001* | |
| Smoking |  |  |  | |  |  |  | |
| No | Reference |  | Reference | |  | Reference |  | |
| Yes | 1.515(1.083-2.121) | 0.015* | 1.560 (0.967-2.515) | | 0.068* | 1.156(0.786-1.700) | 0.461 | |
| Drinking |  |  |  | |  |  |  | |
| No | Reference |  | Reference | |  | Reference |  | |
| Yes | 1.283(0.827-1.991) | 0.267 | 1.293(0.694-2.410) | | 0.418 | 1.236(0.755-2.024) | 0.400 | |
| Treatment |  |  |  | |  |  |  | |
| IMRT | Reference |  | Reference | |  | Reference |  | |
| CCRT | 0.620(0.324-1.183) | 0.147 | 1.454(0.436-4.842) | | 0.542 | 0.640(0.305-1.344) | 0.239 | |
| CCRT+IC/AC | 0.633(0.347-1.157) | 0.138 | 1.204(0.374-3.882) | | 0.756 | 0.676(0.338-1.351) | 0.268 | |
| pre-HGB |  |  |  | |  |  |  | |
| <120g/L | Reference |  | Reference | |  | Reference |  | |
| ≥120g/L | 0.701(0.495-0.994) | 0.046* | 0.892(0.530-1.501) | | 0.668 | 0.691(0.469-1.018) | 0.062* | |
| pre-ALB |  |  |  | |  |  |  | |
| <43g/L | Reference |  | Reference | |  | Reference |  | |
| ≥43g/L | 2.050(1.457-2.885) | <0.001* | 0.397(0.240-0.654) | | <0.001* | 0.530(0.364-0.773) | 0.001* | |
| pre-LMR |  |  |  | |  |  |  | |
| <2.82 | Reference |  | Reference | |  | Reference |  | |
| ≥2.82 | 0.319(0.219-0.462) | <0.001* | 0.346(0.206-0.583) | | <0.001* | 0.291(0.191-0.444) | <0.001* | |
| pre-CRP |  |  |  | |  |  |  | |
| <10mg/L | Reference |  | Reference | |  | Reference |  | |
| ≥10mg/L | 14.246(9.402-21.587) | <0.001* | 9.530(5.604-16.205) | | <0.001* | 0.085 (0.055-0.132) | <0.001* | |
| pre-NLR-PLR |  |  |  | |  |  |  | |
| LRG+ MRG  (0-1 point) | Reference |  | Reference | |  | Reference |  | |
| HRG (2 point) | 3.036(2.178-4.232) | <0.001* | 3.583(2.225-5.770) | | <0.001* | 2.621(1.812-3.792) | <0.001* | |

* indicates statistically significant with p < 0.1.

HR, hazard ratio; CI, confidence interval.

**Supplementary Table 3** Chemotherapy regimens for different groups

| Chemotherapy regimens | Total (%) | pre-NLR (%) | | *p*-Value | pre-PLR (%) | | *p*-Value |
| --- | --- | --- | --- | --- | --- | --- | --- |
|  |  | Low(n=484) | High(n=281) |  | Low(n=446) | High(n=319) |  |
| CCRT |  |  |  | 0.015* |  |  | 0.119 |
| Cisplatin | 580(80.3) | 378(83.1) | 202(75.7) |  | 344(82.3) | 236(77.6) |  |
| Other platinum | 142(19.7) | 77(16.9) | 65(24.3) |  | 74(17.7) | 68(22.4) |  |
| IC |  |  |  | 0.994 |  |  | 0.468 |
| GP | 198(56.0) | 125(56.1) | 73(56.2) |  | 107(55.6) | 91(56.8) |  |
| PF | 50(14.2) | 36(16.1) | 14(10.8) |  | 28(14.5) | 22(13.8) |  |
| TPF | 31(8.8) | 19(8.5) | 12(9.2) |  | 17(8.8) | 14(8.8) |  |
| TP | 74(21.0) | 43(19.3) | 31(23.8) |  | 41(21.2) | 33(20.6) |  |
| AC |  |  |  | 0.438 |  |  | 0.488 |
| GP | 138(76.2) | 92(75.4) | 46(78.0) |  | 82(78.1) | 56(73.7) |  |
| PF | 14(7.7) | 8(6.6) | 6(10.1) |  | 6(5.7) | 8(10.5) |  |
| TP | 29(16.1) | 22(18.0) | 7(11.9) |  | 17(16.2) | 12(15.8) |  |

* indicates a significant difference among groups with *p* < 0.05.

NLR, neutrophil-to-lymphocyte ratio; PLR, platelet-to-lymphocyte ratio; CCRT, concurrent chemoradiotherapy; IC, induction chemotherapy; AC, adjuvant chemotherapy; GP, gemcitabine + cisplatin; PF, cisplatin + 5-fluorouracil; TPF, docetaxel + cisplatin + 5-fluorouracil; TP, docetaxel + cisplatin; pre, pretreatment.
